# Anaerobic membrane bioreactor (AnMBR) with external ultrafiltration membrane for the treatment of sugar beet vinasse

**DOI:** 10.3389/fbioe.2024.1491974

**Published:** 2024-11-20

**Authors:** Beatriz Egerland Bueno, André Luiz Muniz Brito, Victor. S. Garcia Rea, Rifki Wahyu Kurnianto, Marcelo Zaiat, Jules. B. van Lier

**Affiliations:** ^1^ Biological Processes Laboratory, Department of Environmental Engineering, University of Sao Paulo, São Carlos, Brazil; ^2^ Sanitary Engineering Section, Department of Water Management, Delft University of Technology, Delft, Netherlands; ^3^ Department of Sanitary and Environmental Engineering, University of Paraiba State, Campina Grande, Brazil; ^4^ Department of Chemical Engineering, Universitas Gadjah Mada, Yogyakarta, Indonesia

**Keywords:** beet vinasse, ultrafiltration, helix membrane, flux, anaerobic digestion

## Abstract

Vinasse, a by-product of ethanol production, is generated at significant rates. While rich in nutrients such as calcium, magnesium, and potassium, its high solids, organic matter, acidity, and sulfate content pose challenges when disposed directly on soil, necessitating treatment. Anaerobic digestion is a viable solution, reducing organic pollution while recovering energy in the form of biogas, aligning with the biorefinery concept. Traditionally, sludge bed reactors and anaerobic contact reactors are utilized for vinasse processing, with sludge granulation being vital for treatment success. However, challenges such as sludge wash-out due to recalcitrant compounds, high solids concentration in the influent, low pH, salinity, and temperature hinder granule formation. Anaerobic membrane bioreactors (AnMBR) offer an alternative, simplifying treatment by integrating intensified pre- and post-treatment units. Due to complete sludge retention, AnMBRs achieve high COD removal efficiencies, yielding a suspended solids-free and largely disinfected effluent. Therefore, AnMBRs show promise for vinasse treatment, eliminating the need for sludge granulation and producing nutrient-rich effluent with minimal residual organics and suspended solids. In this study, an AnMBR equipped with an inside-out external crossflow ultrafiltration membrane was proposed for the treatment of vinasse. The AnMBR reached a COD removal efficiency of 95% ± 2.6% and produced 0.3 CH_4_ L. g COD _removed_^-1^ working at organic loading rates of 8 g COD. L^-1^ d^-1^ and membrane fluxes of 10 LMH. At organic loading rates of 10 g COD. L^-1^ d^-1^ and fluxes of 12 and 14 LMH, the COD removal efficiency decreased to 77% ± 11% and 73% ± 7.9%, respectively. The AnMBR technology represents an innovation for wastewater treatment, however, more research using the cross-flow configuration and different types of effluents is needed. Literature studies that address the treatment of sugar beet or sugarcane vinasse using AnMBR are still scarce. This study explored the potentials of AnMBR technology for vinasse treatment and contributes to the dissemination of this technology, opening new possibilities for vinasse processing.

## 1 Introduction

Beet vinasse is a sub-product of ethanol production. The beet processing causes environmental problems mainly due to the large production of vinasse and water consumption (Vaccari et al., 2005). Vinasse generation in ethanol distilleries varies between 250 and 500 m^3^ h^-1^ (Junqueira et al., 2017). The substantial volume of vinasse produced underscores the need for effective management and utilization of this by-product. Beet vinasse is rich in nutrients, such as calcium, magnesium, and potassium, making it a potential source of fertilizers (Madejón et al., 2001). However, its composition also presents challenges due to high concentrations of solids, organic matter, and salinity, along with high pH and elevated sulfate content. Therefore, it can affect the productive quality of the soil when it is directly disposed of without previous treatment (Madejón et al., 2001; Fuess and Garcia, 2014).

The application of suitable technologies could lead to the proper use of treated vinasse and side products, such as reuse as industrial process water, use as fertilizer for crop production (beet or sugar cane), and production of a gaseous energy carrier (biogas). Anaerobic digestion is seemingly a proper alternative for the treatment of vinasse to reduce the organic pollution load and simultaneously recover bioenergy, in the form of biogas. Moreover, recent studies highlight the role of anaerobic digestion as a central resource recovery process to enable the use of waste as raw material to produce value-added products and bioenergy by applying a biorefinery concept (Albanez et al., 2016; Fuess et al., 2018).

Thus far, mainly sludge bed reactors and anaerobic contact reactors are used for the anaerobic processing of vinasse. Particularly for sludge bed reactors, sludge granulation is indispensable for the success of the treatment (van Lier et al., 2020). However, sludge wash-out can happen due to the presence of recalcitrant compounds, high levels of solids, off-spec pH values, high salinity, and high temperature; therefore, affecting the process of granule formation (van Lier et al., 2020; Muñoz Sierra et al., 2019).

Anaerobic membrane bioreactor (AnMBR) technology is an alternative to anaerobic contact processes and sludge bed reactor technology, both equipped with intensified pre- and post-treatment units. By operating a single stage mixed reactor system equipped with a membrane unit, a substantial simplification of the treatment train is achieved. Pre-treatment units, such as dissolved air flotation (DAF) and pre-settling, are then not needed anymore and in some cases, post-aeration units can be skipped as well. In addition, AnMBR technology offers complete sludge retention, resulting in very high chemical oxygen demand (COD) treatment efficiencies exceeding 95% and a suspended solids-free effluent that is largely disinfected (Dereli et al., 2012). The AnMBR can be defined as a completely mixed reactor in which anaerobic bioconversion processes occur. The reactor is equipped with a membrane unit that provides highly efficient solids-liquid separation (Lin et al., 2013). Although the technology has been researched for several decades, the sharp drop in membrane prices in past decades resulted in an increasing interest in AnMBR technology which is now being regarded as an innovation in wastewater treatment (Maaz et al., 2019). Nonetheless, to widen its application potential, more research is required on the different reactor configurations and different types of effluents to be treated.

The AnMBR is a promising treatment system for vinasse as it does not require sludge granulation, allows retention of slow-growing microorganisms, minimizes biomass washout, and decouples hydraulic retention time (HRT) from sludge retention time (SRT) (Turker and Dereli, 2021). This results in a final effluent with minimal residual organic content, free of suspended solids (Ozgun et al., 2013), yet rich in nutrients.

Mota et al. (2013) and Santos et al. (2017) investigated the use of a two-stage anaerobic membrane bioreactor (2S-AnMBR), i.e., an acidogenic reactor followed by a methanogenic MBR, equipped with a submerged hollow fiber membrane for the treatment of sugarcane vinasse. In both studies, high COD and sulfate (SO_4_^2-^) removal efficiencies of approximately 97% and 87% were respectively obtained. However, problems with membrane fouling were reported, affecting filtration performance. The membranes had to undergo frequent cleaning routines, meaning that the reactor system had to be stopped to remove and clean the membrane modules.

Using the same two-stage AnMBR configuration, Silva et al. (2020) operated the reactor to evaluate the influence of the COD/SO_4_^2-^ ratio on the performance of vinasse treatment. The AnMBR showed stable performance at the highest ratios with high removal of COD (97.5%) and volatile fatty acids (VFA) (98%) but low removal of sulfate (69.9%), indicating low sulfate-reducing activity. The opposite was observed at lower COD/SO_4_^2-^ ratios, with lower COD and VFA removals but a higher sulfate removal (Silva et al., 2020). Similarly, problems with membrane fouling were reported affecting filtration performance. The submerged reactor configuration required the removal of the membrane module for *ex-situ* cleaning; therefore, reactor operation needed to be stopped. Such procedures might negatively affect process performance and will increase operational costs at full scale.

In AnMBRs with a pressurized external membrane module, the membrane unit is separated from the bioreactor and the membrane operates under pressure to produce the permeate. The suspended anaerobic sludge held in the reactor is pumped into the membrane unit creating a positive pressure that leads to permeate production. Such configuration has several advantages in comparison to the submerged configuration. These advantages include easier hydrodynamic control and higher permeate flows. Additionally, external membrane modules facilitate membrane cleaning and can be easily replaced, allowing anaerobic conditions to be maintained in the main reactor during membrane cleaning or replacement. Because of this, it is easier to remediate membrane fouling in an external cross flow AnMBR in comparison to a submerged configuration (Le-Clech et al., 2006; Lin et al., 2013; Smith et al., 2012).

In recent years, several authors researched vinasse treatment for both water and nutrient recovery using high-retention membrane reactors combined with AnMBR (Silvia et al., 2023). Magalhães et al. (2020) studied the integration of nanofiltration (NF), and reverse osmosis (RO) combined with ultrafiltration (UF) - equipped two-stage anaerobic membrane bioreactor (2S-AnMBR) for the recovery of energy, nutrients (NF/RO concentrate), and water (NF/RO permeate). Silva et al. (2020) evaluated the integration of UF and NF processes to concentrate vinasse and to recover water for reuse. They additionally evaluated the use of UF and NF concentrates for fertigation and the economic viability of the integrated UF-NF system. Carpanez et al. (2022) also evaluated integrated systems to obtain organo-mineral fertilizers, water, and biogas from sugarcane vinasse. They evaluated a combination of a UF-equipped two-stage AnMBR integrated with either RO or NF. Finally, Moreira et al. (2023) studied the potentials of hollow fiber UF membranes for vinasse pre-treatment, aiming to concentrate the vinasse organic matter and to reduce the sulfate content in the retentate for enhancing the methane production potential in subsequent anaerobic digesters.

The primary challenge lies in the efficient treatment of vinasse to obtain a high-quality effluent, which allows for more effective production of fertilizers and the generation of reused water. Thus, the aim of the current study was to evaluate the performance of an AnMBR equipped with an external inside-out ultrafiltration membrane for the treatment of beet vinasse to obtain a high-quality effluent free of suspended solids. Studies using this reactor configuration to treat vinasse are still scarce in the literature. The results of this study may contribute to the future productive use of treated vinasse as industrial process water or fertilizer for crop production.

## 2 Materials and methods

### 2.1 Source of the anaerobic sludge for experimental purposes

Mesophilic anaerobic suspended sludge was obtained from a full-scale side-stream AnMBR treating chocolate products wastewater and used as seed sludge for the AnMBRs. The inoculum sludge was grown under similar shear force conditions and was fully dispersed. In addition, the sludge was already adapted to carbohydrate-degradation. The total suspended solids (TSS) concentration of the seed sludge was 25 ± 0.4 g L^−1^ with a volatile suspended solids (VSS) concentration of 21.85 ± 0.02 g L^−1^.

### 2.2 Vinasse characterization

The vinasse was supplied by a beet biorefinery located in France and stored in 25 L plastic jerrycans in a cold room at −19 °C to avoid biological activity. Before being used, the 25 L jerrycans were transported to a cold room (6°C) for thawing. The vinasse used in the presented research was characterized (Table 1) in terms of COD, VFA, phenol, sulfate, total and volatile suspended solids, pH, sodium (Na), potassium (K), calcium (Ca), and magnesium (Mg).

**TABLE 1:** Vinasse characterization.

Parameter	January 2023 harvest	November 2023 harvest
pH	3.5	3.7
COD raw vinasse (g/L)	24	21.5
COD centrifuged vinasse	23.2	17.8
Sulfate (mg/L)	2091	2,287
Acetic acid (mg/L)	1,059 ± 37	455 ± 9
Propionic acid (mg/L)	17 ± 0.9	20 ± 2
Iso-butyric acid (mg/L)	12 ± 0.3	5 ± 0.03
Butyric acid (mg/L)	6.5 ± 0.4	6 ± 0.4
Iso-Valeric acid (mg/L)	16 ± 0.8	6 ± 0.08
Valeric acid (mg/L)	7 ± 0.03	3.5 ± 0.05
Iso-caproic acid (mg/L)	24 ± 6	21 ± 0.1
Caproic acid (mg/L)	10.5 ± 7	2 ± 1.4
Phenol (mg/L)	5 ± 3	2 ± 0.1
Total suspended solids (g/L)	6	3
Volatile suspended solids (g/L)	5	2.8
Na (mg/L)	104	113
K (mg/L)	1,396	1,501.8
Ca (mg/L)	112	104
Mg (mg/L)	115.5	128

### 2.3 Feed preparation: centrifugation and alkalization

Centrifugation was conducted to simulate the yeast removal step that vinasse undergoes during the process at the beet biorefinery. In the present lab research, the centrifugation was performed at 3,500 rpm for 7 min on a Labofuge 400 Centrifuge at room temperature. After the centrifugation, approximately 70% of the total suspended solids were removed. The raw vinasse was characterized by an acidic pH of 3.54. Therefore, pH corrections were deemed necessary during the reactor’s stabilization period. During the reactors’ start-up, a ratio of 0.7 g NaHCO₃.g vinasse COD^−1^ was used to provide a substrate with pH 7. After the stabilization period, the ratio stepwise decreased to zero.

### 2.4 Experimental setup: AnMBR

The AnMBRs consisted of continuously stirred tank reactors with a volume of 7.0 L and a working volume of 6.5 L connected to external ultrafiltration (UF) polymeric PVDF inside-out membrane modules with 30 nm mean pore size (Pentair, U.S.). The characteristics of this membrane are summarized in Supplementary Table S1.

The temperature in the AnMBR was maintained at 35°C ± 1 °C using a thermostat-water bath (Tamson TC16, NL). The AnMBRs setup was fully automated and controlled, according to Garcia-Rea et al. (2022). The operational parameters such as pressures, volume level, temperature, and pH inside the reactor were collected by the LabVIEW software. The transmembrane pressure (TMP) was calculated in *mbar* based on the pressure at the membrane module inlet, outlet and permeate exit. The calculation was automatically performed by the LabVIEW software. At least three times a week, the feed and permeate pumps were calibrated to check the flow and to guarantee the maintenance of the reactor level. Other membrane parameters were calculated using Equations 1–3:

$$\text{Flux }\left(L.m^{-2}.h^{-1}\right):J=\frac{Q_{\;permeate}}{A_{f}}$$ (1)

$$\text{Permeability }\left(L.\text{mbar}.m^{-2}.h^{-1}\right):L_{p}=\frac{J}{TMP}$$ (2)

$$\text{Resistance }\left(m^{-1}\right):R=\frac{TMP}{J*\;µ}$$ (3)

Where:

*Q_permeate_* is the permeate flow (m^3^.h^-1^).

*A*_*f*_ is the filtration area (m^2^).

µ is the viscosity of the permeate (0.9 mPa s).

Membrane chemical cleaning was performed when the flux could no longer be maintained. The membranes were immersed in a 300-ppm sodium hypochlorite (NaOCl, active chlorine) solution for 3 h to remove organic matter, after which the membrane module was immersed in a 5 g L^-1^ citric acid (C_6_H_8_O_7_) solution for 3 h to remove inorganic matter.

### 2.5 AnMBR operation and sampling

Two experiments were performed in this study. In the first one, two AnMBRs were operated to compare the performance using non-centrifuged and centrifuged vinasse. In this experiment, a non-helix membrane was used. The AnMBRs’ operation was divided into four different phases in which several conditions regarding the organic loading rate (OLR), HRT, and flux (J) were applied (Table 2). The applied cross flow velocity (CFV) through the membrane module was 1 m s^-1^, corresponding to a flow recycle of 1,830 L d^-1^. There was no sludge disposal during the operation of the reactors with the SRT being approximately equal to the operating time. The AnMBRs filtration cycle was as follows: (1) 500 s of filtration, (2) 10 s of backwash, and (3) 10 s of idle.

**TABLE 2:** Operational conditions applied in each phase of the first experiment.

Phases	Days	HRT (d)	Flow rate (L.d^-1^)	OLR (gCOD.L^-1^.d^-1^)	J (LMH)
I	0–39	13	0.5	2	2
II	40–78	6.5	1.0	4	4
III	79–99	4.33	1.5	6	6
IV	100–119	3.25	2.0	8	8
V	120–190	Flux recovery tests			

In the second experiment, one AnMBR was operated using a helix-UF external membrane. Table 3 shows the operational conditions used in this experiment. The helix technology improves the membrane performance by creating turbulence inside the membrane channel; therefore, reducing the fouling rate. In this experiment, the degradation of sugar beet vinasse from different harvests was evaluated using the helix-UF membrane module.

**TABLE 3:** Operational conditions applied in each phase of the second experiment.

Phases	Days	HRT (d)	Flow rate (L.d^-1^)	OLR (gCOD.L^-1^.d^-1^)	J (LMH)
I	0–14	4.3	1.5	4.2	6
II	15–32	3.25	2.0	5.5	8
III	33–97	2.6	2.5	8	10
IV	98–107	2.2	3.0	10	12
V	108–119	1.9	3.5	10	14

Biogas, feed, and permeate samples were collected and analyzed three times a week during both experiments. Permeate and feed samples were analyzed in terms of COD, sulfate, pH, alkalinity, and VFA. COD samples were measured by spectrophotometry using a Hach DR3900 (Hach, Colorado, United States) spectrophotometer. Depending on the COD concentration, LCK314 or LCK514 COD cuvette tests (Hach, Colorado, United States) were used following the manufacturer’s instructions. Hach Lange LCK153 sulfate cuvette tests were used for sulfate determination. Proper dilutions were made to keep the concentrations in the measurable range. VFA and biogas composition were determined as reported in Garcia-Rea et al. (2022). Alkalinity was measured using 0.1 M HCl solution to titrate 50 mL of permeate in an Eco Titrator (Metrohm, Netherlands) following the manufacturers’ instructions. The pH was measured using a pH-meter. Inorganic compounds (Na, K, Mg, Ca) were analyzed using ion chromatography (Eco IC - Metrohm - C6 Cation 150/4.0 Method). Turbidity was measured using a Hach 2100N Turbidimeter, STABLCAL (range: 0–4000NTU). Sludge samples were also collected from the reactor weekly for VSS analyses (Rice et al., 2012) and monthly for microbial community structure analysis before the operational phases were changed. All the analyses were performed in triplicates when possible.

### 2.6 Microbial community dynamics

Sludge samples were regularly collected from the reactors, stored in Eppendorf vials, and centrifuged at 14,000 rpm for 5 min on an Eppendorf Centrifuge 5417R at room temperature. The supernatant was discarded, and the sludge sample was stored at −80°C. Before the DNA extraction, the samples were thawed at 4C°. The DNA was harvested using a DNA extraction kit (FastDNA Spin Kit for Soil, MOP Biomedicals, United States) following the instructions of the manufacturer. The amplification of the DNA (16S rRNA gene) was performed on the Illumina Novaseq 6,000 platform, facilitated by Novagene. The primer set 341 F [(5′-3′) CCTAYGGGRBGCASCAG] and 806 R [(5′-3′) GGACTACNNGGGTATCTAAT] were utilized to amply the hypervariable regions V3-V4. The polymerase chain reaction (PCR) procedures were conducted using Plusion High-Fidelity PCR master Mix, manufactured by New England Biolabs.

The sequencing reads were subjected to quality filtering and denoising, with the amplicon sequence variants (ASVs) identified using the DADA2 plugin (Callahan et al., 2016). Chimeric sequences were removed through the “consensus” method. Taxonomic classification of the representative ASV sequences was performed using the “classify-consensus-vsearch” plugin (Rognes et al., 2016), referencing the SILVA (132) database. The exemplar sequences were aligned via the MAFFT algorithm (Katoh and Standley, 2013), and a phylogenetic tree was constructed using FastTree (Price et al., 2010). The feature table and tree were then imported into the R environment. Differential abundance analysis between different reactor operation stages was conducted using the DESeq2 library (Love et al., 2014). The phyloseq library (McMurdie and Holmes, 2013) was employed for visualizing the abundance data and phylogenetic tree. The sequences in this study could be accessed in the NCBI database under the accession number PRJNA1148776.

## 3 Results and discussion

### 3.1 AnMBR performance treating non-centrifuged and centrifuged vinasse

#### 3.1.1 Monitoring analysis

**FIGURE 1:**
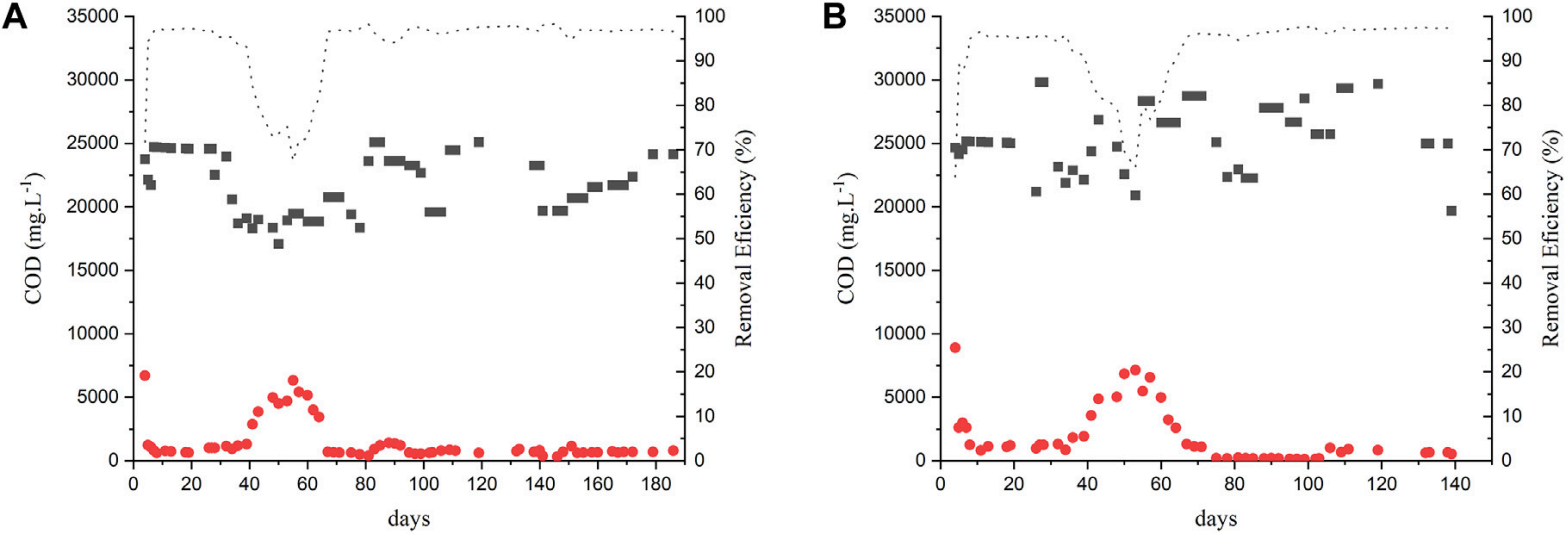
COD removal in experiment 1: **(A)** AnMBR1-Centrifuged vinasse; **(B)** AnMBR2-Raw vinasse. 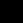 Influent; 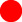 Effluent; 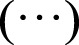 removal efficiency.

The reactors were seeded with approximately one-third of the reactor’s volume as seed sludge. The VSS had an initial concentration of 7.44 gVSS.L^-1^. The sludge of both reactors was adapted for approximately 40 days (Phase I) with an OLR of 2 kg COD. m^-3^.d^-1^. After this period, the OLR was stepwise increased after COD removal efficiencies above 90% were achieved. Figures 1A, B show the COD removal efficiencies of the AnMBR 1 and 2, respectively, along their operation. As the AnMBR effluent samples passed through the membrane with a nominal pore size of 30 nm (i.e., < 0.45 μm pore size for filtered samples), it was not necessary to perform soluble COD analysis. Thus, from a technological point of view, the reactors were compared in terms of total COD.

The COD removal efficiency in both reactors remained above 95% except during days 40 and 60 when the first OLR increase was applied, with concomitant bicarbonate reduction. The OLR was increased according to Table 2. Studies reporting the anaerobic treatment of vinasse using other types of reactors obtained lower COD removal efficiencies working with similar or higher OLR. Aquino et al. (2017) reported a COD removal efficiency of 86% ± 3.2% treating sugarcane vinasse in a fixed-bed anaerobic reactor at OLR of 5.5 kg COD.m^-3^.d^-1^ and 84% ± 1.3% at OLR of 10.2 kg COD.m^-3^.d^-1^. In another study, Fuess et al. (2018) performed sugarcane vinasse treatment in an acidogenic reactor followed by an upflow anaerobic sludge blanket reactor (UASB). OLRs of 15, 20, and 25 kg COD. m^-3^.d^-1^ were applied and COD removal efficiencies of up to 70% were achieved. Mota et al. (2013) and Santos et al. (2017) used two-stage submerged anaerobic membrane bioreactor for the treatment of sugarcanne vinasse. The studies reported similar COD removal efficiencies of approximately 97% at OLRs of 2.5 and 6 g COD.L^-1^.d^-1^, respectively.

**FIGURE 2:**
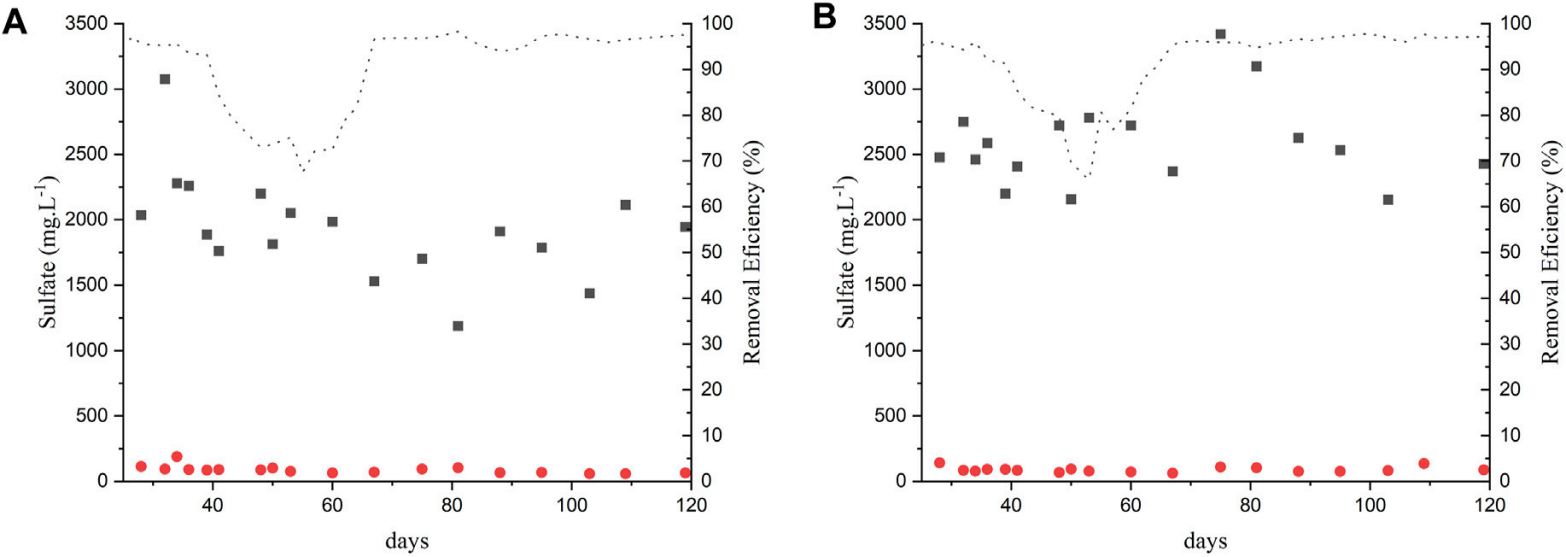
Sulfate removal in experiment 1: **(A)** AnMBR1-Centrifuged vinasse; **(B)** AnMBR2-Raw vinasse. 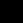 Influent; 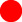 Effluent; 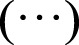 removal efficiency.

Sulfate removal was observed in both reactors, indicating the occurrence of sulfate reduction. Figures 2A, B show the sulfate removal during the operation of AnMBR1 and 2. The sulfate removal was higher than 70%, surpassing 95% during the last phases of operation. In the present study, the applied COD/SO_4_^2-^ ratio was ≈10 and high COD and high sulfate removal efficiencies were obtained unlike what was reported in previous studies (Silvia et al., 2010). Quantification of the H_2_S content in the biogas was not possible due to a lack of analytical equipment.

Sulfate reducing bacteria (SRB) reduce sulfate to sulfide that can dissolve in the effluent (HS^−^, S^2-^, H_2_S) or becomes part of the biogas (H_2_S) (Jimenez et al., 2017). Sulfide generation was monitored on the permeate since Phase II. It is assumed that the sulfide concentration in the reactor was the same as in the permeate as it is not anaerobically converted. The sulfide concentration observed in AnMBR1 was 96.2 ± 20.6 mg.L^-1^, 104 ± 0.8 mg.L^-1^ and 103.2 ± 0.01 mg.L^-1^ during Phases II, III and IV, respectively. In AnMBR2 the sulfide concentrations were 127 ± 0.4 mg.L^-1^, 81 ± 23.7 mg.L^-1^ and 52.4 ± 12 mg.L^-1^, at Phases II, III and IV, respectively.

The presence of sulfate stimulates SRB populations, reducing the methanogenesis because of competition for substrates (Fuess and Garcia, 2015). Methanogenesis is affected by the COD/SO_4_^2-^ ratio (Sarti and Zaiat, 2011); at low ratios sulfate reduction predominates, while methanogenesis prevails at higher values (Madden et al., 2014). In the present study, with a COD/SO_4_^2-^ ratio of 10, sulfide was detected in the permeate; however, the COD removal efficiency was not affected. Different threshold concentrations for H_2_S inhibition in literature can be found as the sulfide inhibition in anaerobic reactors depends on several factors such as pH, substrate characteristics, COD/SO_4_^2-^ ratio, presence of metals, organic and hydraulic load, sludge type and biomass acclimation (Turker and Dereli, 2021).

VFA was monitored weekly. The results showed a similar trend as the COD concentrations. A maximum of 90% reduction in VFA concentration was observed in the permeate of the reactors (Supplementary Figure S1). Acetic acid accumulation was observed in AnMBR1 and AnMBR2 during the adaptation phase and the first OLR increase indicating an impairment of the methanogenic activity (Supplementary Figure S2). Propionic acid accumulation was observed in the AnMBR2 during the first days of operation and it could be related to an unbalance in the acetogenesis step, which is expected to occur at the start-up phase of an anaerobic process. During the first increase in OLR (days 40–60) the VFA removal efficiencies dropped due to acetic and propionic acid accumulation. As discussed previously, the increase in OLR and the decrease in bicarbonate supply likely caused stress in the sludge. It took approximately 20 days to get both reactors stabilized again. Interestingly, during subsequent OLR increases, the reactor performance remained stable and no VFA accumulation was observed which could be related to the adaptation and/or selection of the microbial community. In subsequent periods including OLR increments, the VFA concentration in the permeate remained below 250 mg. L^-1^ with VFA concentrations reaching almost 0 mg. L^-1^, mainly after the Phase II.

During the operation of both reactors, the pH of the feed, permeate and the reactors’ matrix were continuously monitored (Supplementary Figure S3). Due to the low pH of the vinasse, NaHCO_3_ was used to neutralize the influent during the acclimation period at a ratio of 0.7 g bicarbonate. g^-1^COD. After this period, the alkalinity was weekly measured (Supplementary Table S2) and the ratio was decreased to 0.3 g bicarbonate. g^-1^COD and finally NaHCO_3_ was completely removed from the influent. The pH in the reactor was maintained approximately at 7.0 during the whole operational period. Even when bicarbonate was no longer supplied, the reactor pH remained stable at neutral values.

The VSS concentration in both reactors was monitored during the reactors’ operation (Supplementary Figure S4). No sludge disposal was performed during the operation with the SRT being approximately equal to the operating time. The VSS concentration gradually increased in both reactors after the adaptation period and the first OLR increase, indicating sludge growth. At the end of the operation the VSS concentration in the AnMBR1 was 39 ± 0.03 g VSS. L^-1^ and in the AnMBR2 was 49 ± 0.02 g VSS.L^-1^. AnMBR2 presented higher VSS concentrations than AnMBR1, which might be attributable to the fact that 70% of the solids in the feed of AnMBR1 were reduced after centrifugation of the influent vinasse. Apparently, another (non-active) fraction of organic solids was accumulating in AnMBR2.

The biogas production and composition measured during the different stages of the reactor’s operation can be found in the (Supplementary Table S3). The biogas production was very similar in both reactors except in Phase IV when AnMBR2 presented lower biogas (12 ± 6 L d^-1^) production than AnMBR1 (27 L d^-1^), probably due to the operation issues observed during that phase (Section 3.2.1). The methane yield observed remained around 0.28 CH_4_ L. g COD_removed_^-1^ in AnMBR1 and 0.24 CH_4_ L. g COD_removed_^-1^ in AnMBR2.

#### 3.1.2 Membrane performance

**FIGURE 3:**
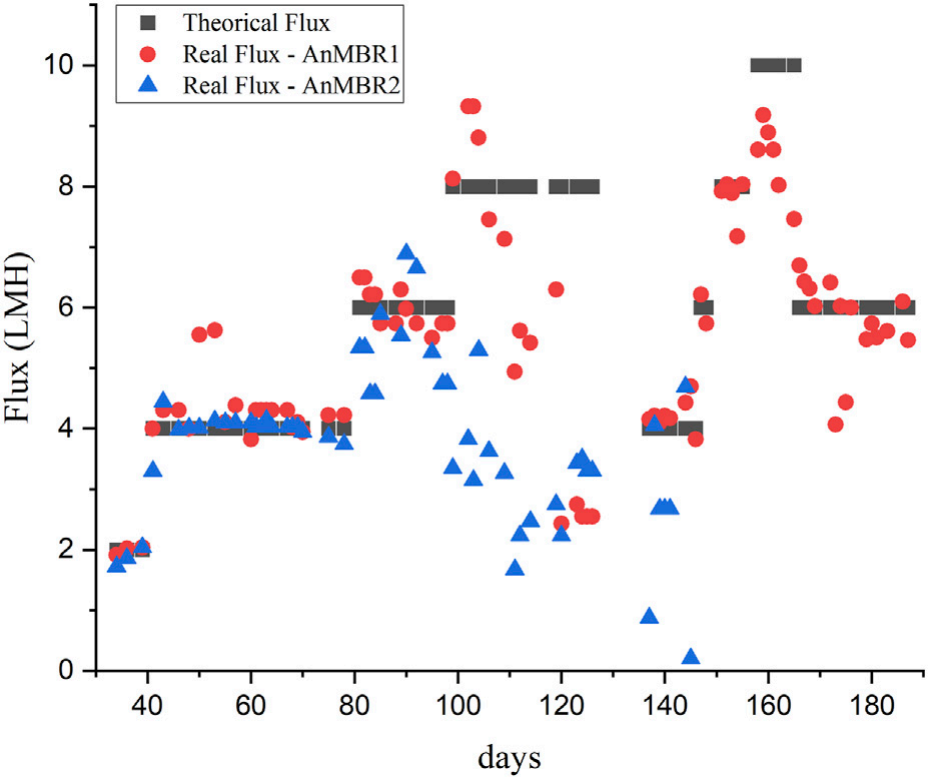
Theoretical and real membrane flux monitored during experiment 1.

Figure 3 shows membrane flux monitored during the reactor’s operation. Other membrane parameters can be found in the Supplementary Figure S5.

The flux of the AnMBR2 began to decline after Phase III, at a flux of 6 LMH, as illustrated in Figure 3. Notably, filtration resistance (Supplementary Figure S5) started to rise on day 80, concomitant with the flux increase to 6 LMH. The resistance continued increasing despite the chemical cleaning procedure before the flux was increased to 8 LMH to restore membrane permeability, leading to complete membrane clogging during Phase IV. The flux was decreased to 4 LMH and the crossflow velocity was increased from 1 m s^-1^–2 m s^-1^ after performing the membrane cleaning procedure. However, despite the cleaning procedures, the membrane permeability did not recover (Supplementary Figure S5), the flux was not recovered and the reactor showed process instability and subsequent membrane clogging. The observed decreased performance of the membrane prompted the decision to cease the reactor operation. It's noteworthy that COD removal efficiencies remained unaffected by these membrane-related operational issues during that period, as depicted in Figure 1B.

AnMBR1’s flux started to decrease after the increase to 8 LMH, in Phase IV (Figure 3). The highest values for the filtration resistance were observed on day 120 (Supplementary Figure S5). Subsequently, a chemical cleaning procedure was performed, the CFV was increased to 2 m s^-1^, and the system was restarted at a flux of 4 LMH followed by an incrementally raise to 8 LMH. A tentative increase to 10 LMH resulted in the membrane clogging. The use of centrifuged vinasse showed a better filtration performance as it contained fewer solids that contribute to the membrane fouling.

#### 3.1.3 Microbial community dynamics

**FIGURE 4:**
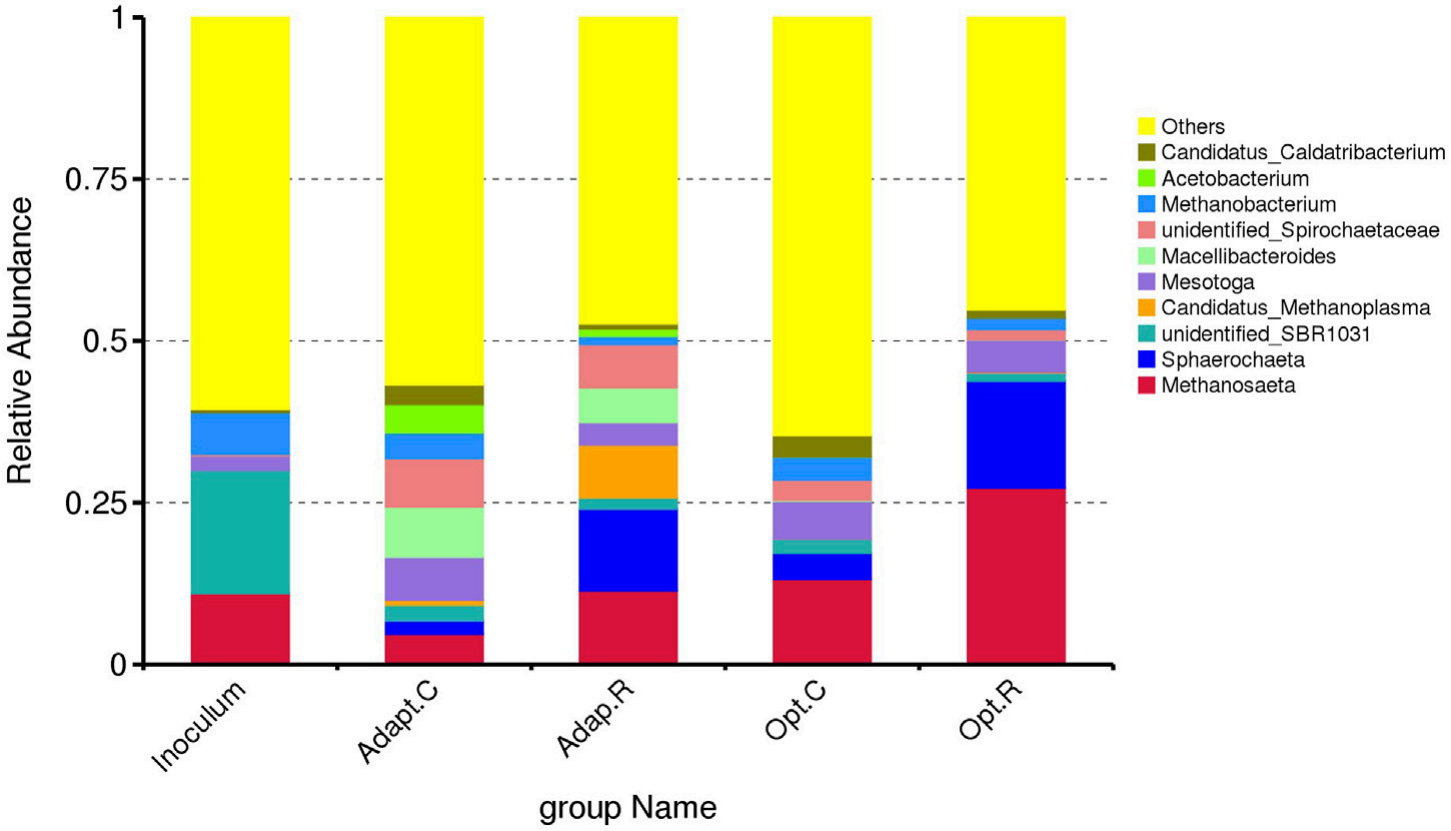
Comparison between the top genera with relative abundance higher than 1% in the inoculum and sludge from AnMBR1 after Phase I (Adapt. C) and Phase III (Opt.C) and from AnMBR2 after Phase I (Adapt. R) and Phase III (Opt.R).

Figure 4 shows the microbial composition at genera level of the inoculum and sludge samples collected from the AnMBR 1 and 2 at the end of Phases I and III. Phase I was the adaptation phase, in which the inoculum had the first contact with the vinasse. Phase III was chosen to be analyzed, because it was the phase in which the highest removal efficiencies (95%) were achieved, and the membrane flux could be maintained (6 LMH).

The inoculum presented high relative abundance of *Methanosaeta,* unidentified*-SBR1031* and *Methanobacterium*. *Methanosaeta* is an acetate-converting methane-producing archaea and one of the key populations in methanogenic bioreactors. Most of the COD is eventually converted via acetate to methane, agreeing with the pre-dominant relative abundance of the acetoclastic *Methanosaeta* in the AnMBR sludge of our present study. On the other hand, species belonging to the genus *Methanobacterium* are hydrogenotrophic methanogens that utilize H₂ and CO₂ as substrates for methane production (Whitman, et al., 2014). Their ability to grow autotrophically under strict anaerobic conditions makes them integral to the hydrogenotrophic methane pathway, complementing the acetoclastic pathway of *Methanosaeta*. The unidentified *SBR1031* group appears to play a role in acetogenic dehydrogenation, potentially using ethanol as a carbon source (Xia et al., 2016).

After the adaptation period, the relative abundance of both unidentified *SBR1031* and *Methanobacterium* decreased in both reactors, while the relative abundance of *Methanosaeta* decreased only in AnMBR1, suggesting distinct community dynamics between the reactors. Aditionally, in AnMBR1, other microbial populations of interest emerged: *Mesotoga*, *Macellibacterioides*, unidentified *Spirochaetaceae,* and *Acetobacterium*.

*Mesotoga*, a mesophilic acetogen, is common in hydrocarbon-rich anaerobic environments. Besides being mesophilic, *Mesotoga* displays lineage-specific phenotypes, such as no or little H_2_ production and dependence on sulfur-compound reduction, which may influence its ecological role (Nesbø et al., 2019). In this sense, *Mesotaga* may have influenced the system by reducing sulfur compounds rather than producing H₂, which could impact electron flow in the bioreactor. *Mesotoga* was found in all the other AnMBR sludge samples analyzed in this study. *Macellibacteroides*, a fermentative bacterium, produces lactic, acetic, and butyric acids as fermentation products, contributing to the pool of substrates available for methanogenesis (Jabari et al., 2012).

*Acetobacterium* plays an important role as an acetogen, reducing CO₂ to acetate and other multiple-carbon compounds using H₂ as an electron donor, further supporting the acetate-driven methane production pathway (Balch et al., 1977). Interestingly, after adaptation, AnMBR1 exhibited a high relative abundance of *Candidatus Methanoplasma*, an obligate anaerobe that produces methane via methylotrophic metabolism, relying on methyl donors such as methanol and monomethylamine and hydrogen dependent for growth (Lang et al., 2015).

The genera unidentified *Spirochaetaceae* was found in both AnMBRs after adaptation. The family *Spirochaetaceae* comprises a wide range of phylogenetically diverse genera, including *Alkalispirochaeta, Marispirochaeta, Ocean ispirochaeta, Pleomorphochaeta, Rectinema, Salinispira, Sediminispirochaeta, Sphaerochaeta, Spirochaeta, Spironema, Treponema,* and several genera containing uncultured species. Among these, *Sphaerochaeta* was detected in both AnMBRs, though with higher relative abundance in AnMBR2.

*Sphaerochaeta* comprises free-living, chemotrophic, organotrophic, anaerobic, mesophilic, and neutrophilic bacteria with fermentative metabolism. These bacteria have been isolated from various anaerobic environments, such as freshwater sediments, termite hindguts, and methanogenic consortia (Bidzhieva et al., 2020). Fermentative growth is observed with carbohydrates including pentose and hexose monosaccharides, disaccharides, and soluble starch. End products of glucose fermentation include acetate, formate, and ethanol and growth is stimulated by yeast extract (Ritalahti et al., 2012), which could explain its higher abundance in AnMBR2 treating non-centrifuged vinasse. This suggests that *Sphaerochaeta* plays an important role in breaking down complex organic matter in the system, contributing to the pool of fermentation products that feed into methane production. The presence of this genus, alongside other fermentative and methanogenic organisms, highlights its role in the overall process of anaerobic digestion by maintaining metabolic flexibility and supporting the conversion of organic matter into biogas.

An increased relative abundance of the genera *Methanosaeta*, *Sphaerochaeta,* and *Mesotoga* was observed in both reactors in the samples collected in Phase III. The relative abundance of other genera decreased or remained the same. This community composition highlights a complex interplay between acetoclastic, hydrogenotrophic, and methylotrophic methanogens, alongside acetogens and fermentative bacteria, each contributing to the overall stability and efficiency of the anaerobic digestion process. The obtained results showed that the communities developed in both reactors are capable of degrading beet vinasse. Similar microorganisms were found in both reactors, although in different relative abundances.

### 3.2 AnMBR with helix ultrafiltration external membrane performance treating centrifuged vinasse

#### 3.2.1 Monitoring analyses

The reactor was inoculated with 3.4 L of the sludge from the reactor treating centrifuged vinasse from AnMBR1 (39 gVSS.L^-1^). Resulting in an initial VSS concentration of approximately 22 g.L^-1^. The AnMBR2 was fed with the centrifuged beet vinasse. The specific methanogenic activity (SMA) of the sludge used to inoculate each reactor was 0.25 g COD-CH_4_. g^-1^VSS.d^-1^. As the reactor had an initial solid concentration of 22 g VSS. L^-1^, the expected COD conversion rate was 36 g COD.d^-1^, which corresponded to an OLR of 5.5 g COD. L^-1^d^-1^. It was then decided to start the reactor with an OLR below that value and the OLR was stepwise increased after COD removal efficiencies above 90% were achieved, according to Table 3.

**FIGURE 5:**
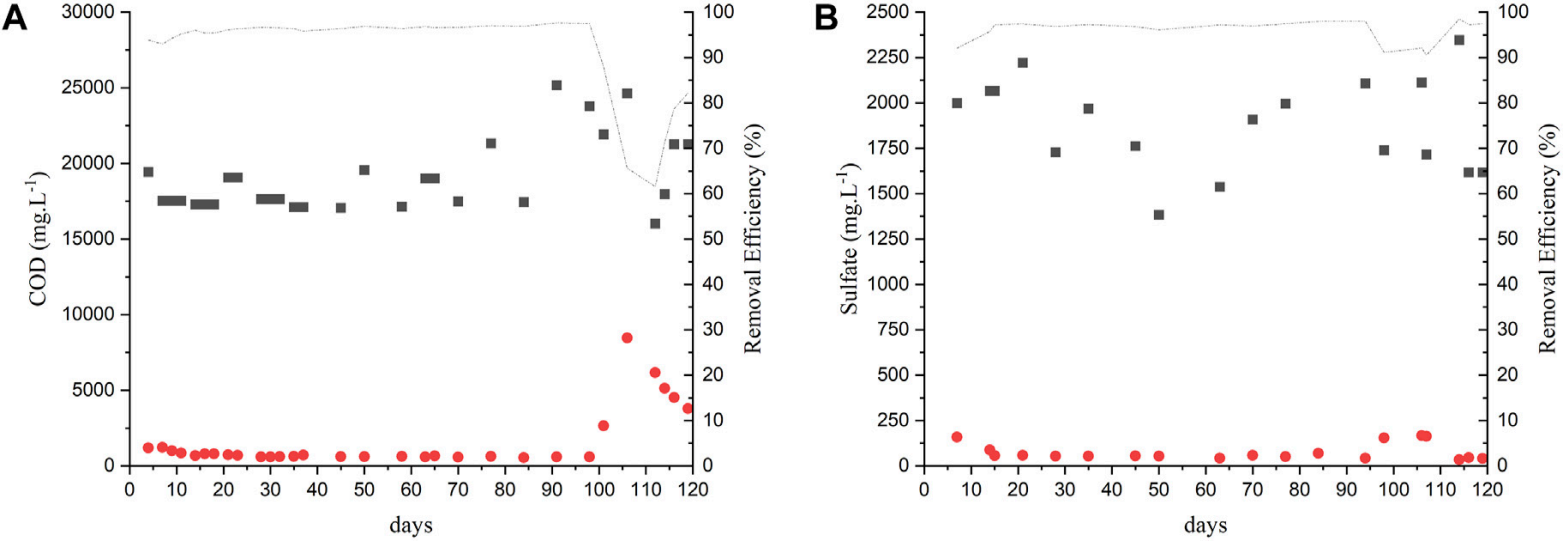
Monitoring AnMBR in experiment 2: **(A)** COD **(B)** sulfate removal efficiency. 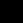 Input; 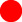 Output; 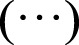 removal efficiency.

The COD removal efficiency was above 95% from day 0 to day 97 of operation (Figure 5A). On day 98 the COD removal efficiency started to be decreased after the OLR was increased to 10 kg COD. m^-3^.d^-1^ and flux to 12 LMH. The COD removal efficiency decreased to its lowest value (62%) on day 110 and then started to increase again, reaching 80% at the end of the reactor’s operation.

Published research using other reactor configurations for the sugarcane vinasse treatment resulted in lower or equal removal efficiencies at the same OLR as applied in this study (Aquino et al., 2017; Fuess et al., 2018). However, the current studies reporting the use of AnMBR for the treatment of sugarcane vinasse did not reached OLRs higher than 6 g COD.L^-1^.d^-1^ (Mota et al., 2013; Santos et al., 2017). Turker and Dereli, (2021) investigated the performance of a pilot scale AnMBR treating beet molasses based industrial wastewater. The reactor achieved a COD removal efficiency between 48% and 92% up to a volumetric load of 10 g COD.L^-1^.d^-1^. When the system operated at an OLR of 10 g COD.L^-1^.d^-1^ the COD removal was approximately 60%.

Biogas production and composition obtained in each phase of the second experiment are presented in Supplementary Table S4. The results are similar to those obtained in the first experiment. Biogas production increased while the CH_4_ portion decreased with the OLR increasing. The low methane content in the biogas produced from vinasse anaerobic degradation is primarily attributed to its complex composition (Fuess and Garcia, 2015; Parsaee et al., 2019) which promotes high acidification in the reactor, especially in a one-stage reactor configuration. The organic acids (normally produced at higher rates by fermenting bacteria than consumed by methanogenic archaea) lower the medium pH and might inhibit the methanogenesis, (Silva et al., 2020). This occurs especially because the AnMBR used in this study is a one-stage (methanogenic) reactor. Two-stage anaerobic reactors provide better conditions for the separate development of acidogenic and methanogenic microorganisms, especially in the treatment of high OLR and low pH effluents, such as vinasse in which acidifying conditions can be clearly separated from the methanogenic ones (Fuess et al., 2017; Santos et al., 2017).

Sulfate removal efficiencies (Figure 5B) remained above 95% throughout the entire operation, except on days 90–105, when the removal efficiency decreased to 90%, probably due to the OLR increase. Mota et al. (2013) and Santos et al. (2017) obtained sulfate removal efficiencies of approximately 87%, using a two-stage membrane anaerobic reactor at OLR lower than the one applied in this study.

VFA were monitored weekly and showed a similar trend as the COD (Supplementary Figure S6). A VFA removal efficiency of 90% was observed in the permeate when compared to the inlet of the AnMBR. The exception occurred after the OLR increased to 10 kg COD. m^-3^. d^-1^ in phases IV and V (from day 98–119), when mainly acetic and propionic acid were accumulated, indicating an unbalance in the acetogenic and methanogenic steps. In general, the VFA concentration in the permeate remained below 100 mg. L^-1^ in almost the entire reactor’s operation.

#### 3.2.2 Membrane performance

**FIGURE 6:**
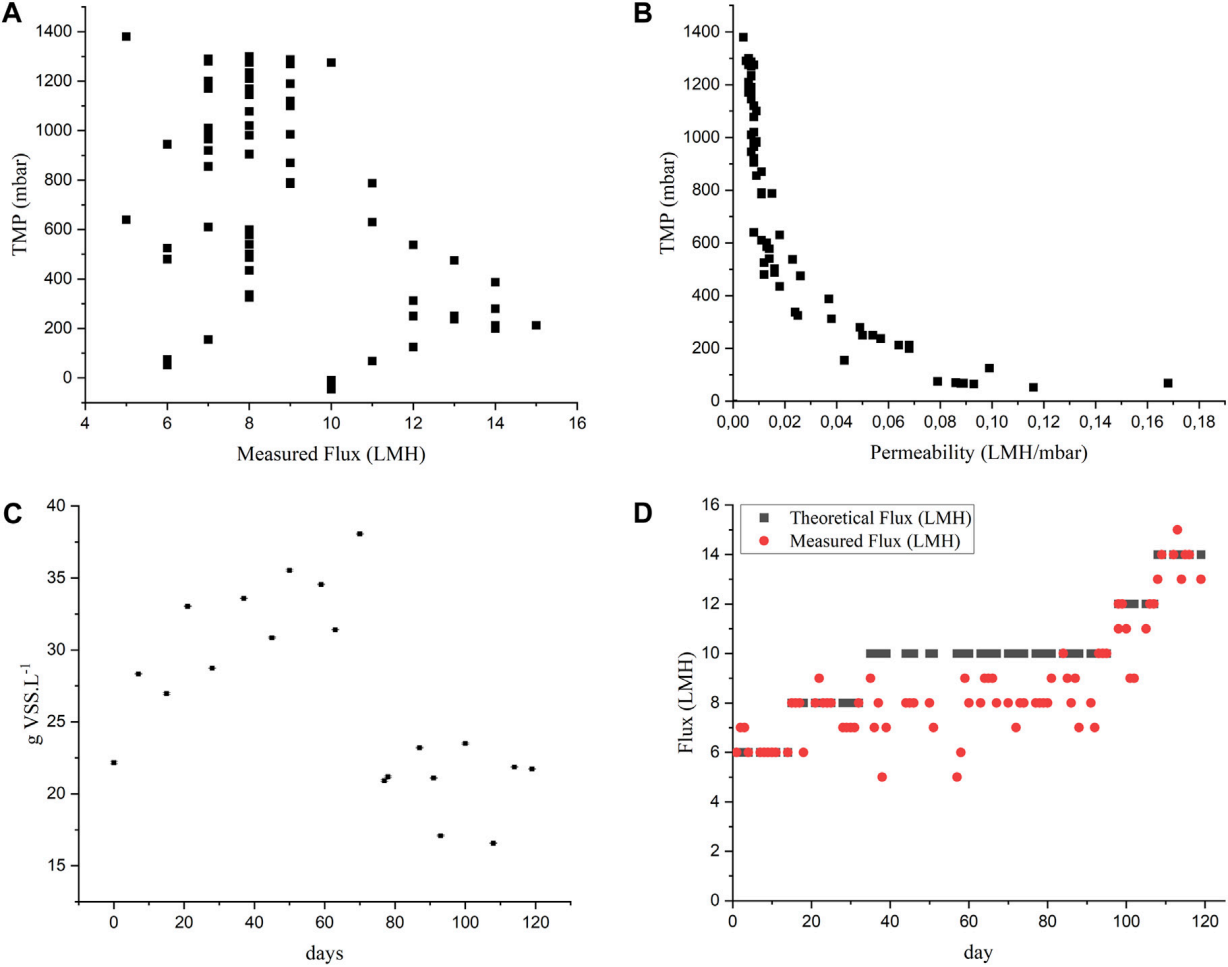
Membrane performance parameters: **(A)** solids concentration in the reactor n = 3, bars = standard deviation, **(B)** the theoretical and real flux, **(C)** transmembrane pressure *versus* flux, **(D)** permeability *versus* transmembrane pressure.

Different strategies to achieve higher fluxes were tested, such as the increase in CFV and the decrease in VSS concentration in the reactor. Figure 6A shows the concentration of solids in the reactor during operation. In Figure 6B, the theoretical flux (flux applied in the system) and the measured flux are compared. Figure 6C shows the variation in TMP according to the membrane flux. Figure 6D illustrates how the permeability and TMP correlated during reactor operation.

In Phases I (days 1–14) and II (days 15–32), a flux of 6 and 8 LMH were applied, respectively. There were no problems maintaining the intended flux in the membrane (Figure 6B). However, when the theorical flux was increased to 10 LMH in phase III, a decrease in the flux and an increase in the TMP were observed. Therefore, on day 39, it was decided to test the effect of increasing the CFV from 1 to 2 m/s. However, there were no improvements observed in the filtration process. Hereafter, it was decided to reduce the VSS concentration in the reactor from 38 to 21 g VSS.L^-1^ on day 72 (Figure 6A). Despite a decrease in the TMP, there were no improvements in the flux. As the COD removal efficiency remained high (≈ 95%), it was decided to further reduce the solids concentration to 17 g VSS. L^-1^ (day 93). Due to technical complications, the CFV was reduced from 2 to 1.5 m/s. Those changes did not affect the COD removal efficiency (Figure 5A) and the eventual measured flux achieved 10 LMH. The TMP reduced significantly due to the decrease in VSS concentration and the membrane cleaning procedures (Figure 6C).

On day 98, the membrane flux was then increased to 12 LMH; however, a decrease in the COD removal efficiency was observed. Nevertheless, on day 110, the flux was further increased to 14 LMH (Phase V) since the main objective of this experiment was to evaluate the filtration efficiency of the membrane at higher fluxes despite the bioconversion performance.

The sludge was continuously wasted to maintain the concentration between 16 and 17 g VSS.L^-1^ to keep the TMP below 1,000 mbar (Figure 6C). The OLR to the reactor did not change despite the increase in the flux due to a lower concentration of the vinasse used in comparison to the vinasse used in Phase IV (Table 3). At this stage, the results showed that a flux of 14 LMH could be maintained; however, the COD removal efficiency dropped to values close to 60%. Using 2S-AnMBR with submerged membrane Mota et al. (2013) and Santos et al. (2017) applied flux of 4.4 and 5.1 LMH, respectively. In other study, Mota et al. (2019) evaluated the anaerobic treatment of a synthetic acidified effluent using an AnMBR with the same configuration as the present study with a flux set as 9.8 LMH. Flux of 14 LMH was also achieved using an AnMBR consisting of an upflow anaerobic bioreactor coupled to two side-stream ultrafiltration membrane modules connected in parallel treating food wastewater (Ariunbaatar et al., 2021). However, no studies about vinasse treatment in AnMBR in laboratory scale have been published yet applying flux higher than 10 LMH.

It was then decided not to further increase the membrane flux so that the associated OLR would not cause the reactor to collapse. In the last days of operation, a trend towards increased COD removal efficiency was observed. It was not possible to determine whether the efficiencies would again reach similar values as those observed until phase III (96%). However, most likely after a longer period of operation under those conditions, the reactor would have shown higher efficiencies.

The results show that reducing the VSS concentration to 16–17 g/L is essential for reducing the TMP, which leads to higher permeability (Figure 6D) and consequently, the maintenance of the desired flux.

## 4 Conclusion

The AnMBR had COD removal efficiencies higher than 95% generating an effluent free of solids and with a neutral pH. Biogas production reached values of 27 L d^-1^ and 0.3 CH_4_. L. g COD _removed_^-1^. In terms of anaerobic biodegradability, both reactors, with centrifuged vinasse and non-centrifuged vinasse, presented a similar performance. However, the AnMBR treating centrifuged vinasse showed a better membrane performance in terms of filtration as it had fewer solids that could contribute to the membrane fouling. The non-helix membrane had maximum attainable fluxes of 6 LMH at a CFV = 1 m/s and 8 LMH at a CFV = 2 m/s.

The AnMBR operation with a helix membrane and a VSS concentration of 16–17 g.L^-1^ showed a maximum attainable flux of 10 LMH with a corresponding COD removal efficiency of 95% ± 3%. A flux of 12 LMH had a corresponding COD removal efficiency was 77% ± 11%. And a flux of 14 LMH had a corresponding COD removal efficiency of 73% ± 8%.

The use of AnMBR with an external ultrafiltration configuration led to a final effluent characterized by reduced COD concentration and devoid of solids while retaining the valuable nutrient content. This approach has strong potential for the transformation of vinasse into a resource that aligns with sustainable and environmentally conscious practices.

The application of AnMBR represents a step towards unlocking the full potential of vinasse as a valuable resource in the realms of industrial water reuse, nutrient acquisition, and (bio)energy recovery. The integration of AnMBR offers a new perspective on how to effectively manage and utilize by-products from ethanol production for a more sustainable and resource-efficient future.

## Data Availability

The datasets presented in this study can be found in online repositories. The names of the repository/repositories and accession number(s) can be found in the article/Supplementary Material.
