# Supplementary material for: Anaerobic membrane bioreactor (AnMBR) with external ultrafiltration membrane for the treatment of sugar beet vinasse
Source: Front Bioeng Biotechnol. 2024 Nov 20;12:1491974. doi: 10.3389/fbioe.2024.1491974 (PMC11615572; doi:10.3389/fbioe.2024.1491974)
Supplement: Supplementary file 1 [file DataSheet1.PDF]

Supplementary Material

S1 Membrane Characteristics

The AnMBRs consisted of CSTR reactors connected to external ultra-filtration (UF) polymeric PVDF inside-out membrane modules with 30 nm mean pore size (Pentair, U.S.). The characteristics of this membrane are summarized in Table S1.

Table S1. Membrane Characteristics

Parameter	Value
Nominal pore size (nm)	30
Type	Tubular, inside out/ non-helix and helix
Brand	Pentair compact 33
Material	PVDF
Diameter (mm)	5.2
Length (cm)	64
Cross sectional area (m^2)	2.1237E-05
Surface area (m^2)	1.0455E-03

S2 Volatile Fatty Acids - Experiment 1

VFA were monitored weekly. The results showed a similar trend as the COD concentrations. A maximum of 90% reduction in VFA concentration was observed in the permeate of the reactors (Figure S1). Acetic acid accumulation was observed in AnMBR1 and AnMBR2 during the adaptation phase and the first OLR increase indicating an impairment of the methanogenic activity (Figure S2). Propionic acid accumulation was observed in the AnMBR2 during the first days of operation, and it could be related to an unbalance in the acetogenesis step, which is expected to occur at the start-up phase of an anaerobic process. During the first increase in OLR (days 40-60) the VFA removal efficiencies dropped due to acetic and propionic acid accumulation. As discussed previously, the increase in OLR and the decrease in bicarbonate supply likely caused stress in the sludge. It took approximately 20 days to get both reactors stabilized again. Interestingly, during subsequent OLR increases, the reactor performance remained stable and no VFA accumulation was observed which could be related to the adaptation and/or selection of the microbial community. In subsequent periods including OLR increments, the VFA concentration in the permeate remained below 250 mg. L^{-1} during the remainder of the experiment, with VFA concentrations reaching almost 0 mg. L^{-1}, mainly after the Phase II.

A **B**

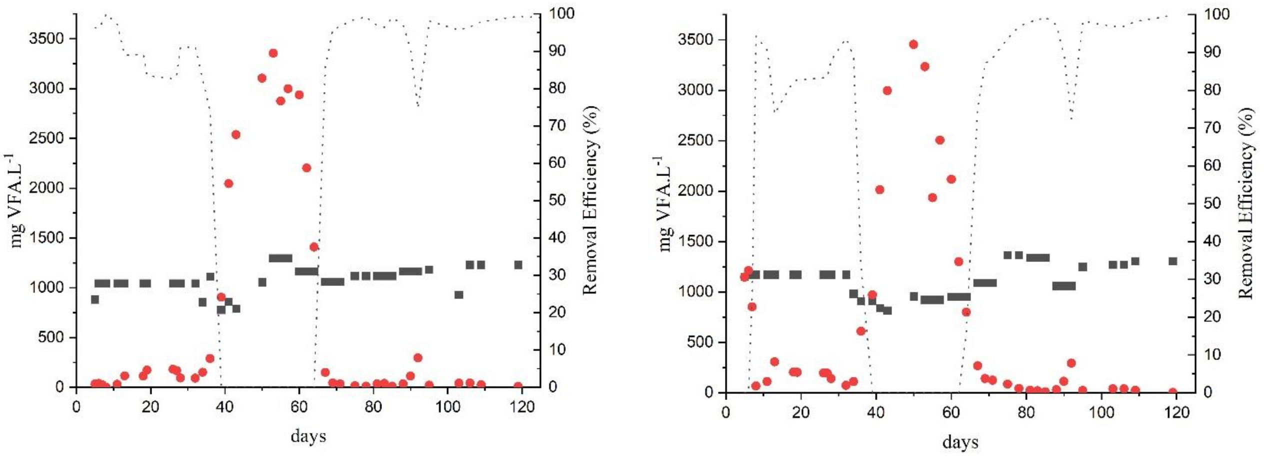

Figure S1. VFA removal efficiency during the AnMBRs operation in Experiment 1: (**A**) AnMBR1-Centrifuged vinasse; (**B**) AnMBR2-Raw vinasse. ■ Influent; ● Effluent; (···) removal efficiency

A **B**

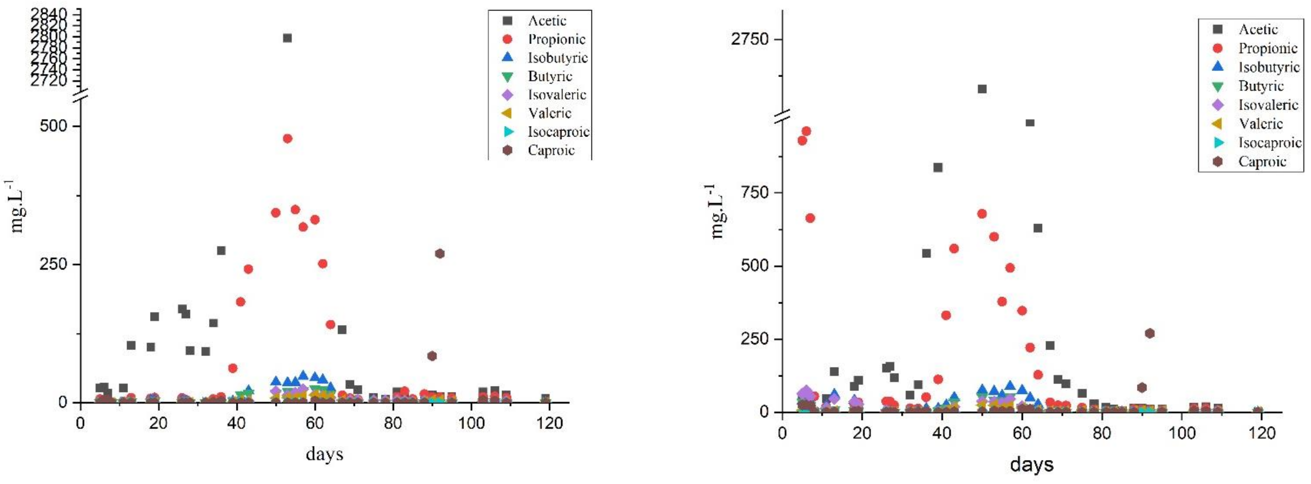

Figure S2. VFA Generation during the AnMBRs operation in Experiment 1: (**A**) AnMBR1-Centrifuged vinasse (**B**) AnMBR2-Raw vinasse

S3 pH and alkalinity monitoring - Experiment 1

During the operation of both reactors, the pH of the feed, permeate and the reactors’ matrix were continuously monitored (Figure S3). Due to the low pH of the vinasse, $NaHCO_3$ was used to neutralize the influent during the acclimation period at a ratio of 0.7 g bicarbonate. g^{-1}COD. After this period the alkalinity was weekly measured (Table S2), and the ratio was decreased to 0.3 g bicarbonate. g^{-1}COD and finally $NaHCO_3$ was completely removed from the influent. The pH in the reactor was maintained approximately in 7.0 during the whole operational period. Even when bicarbonate was no longer supplied, the reactor pH remained stable at neutral values.

A **B**

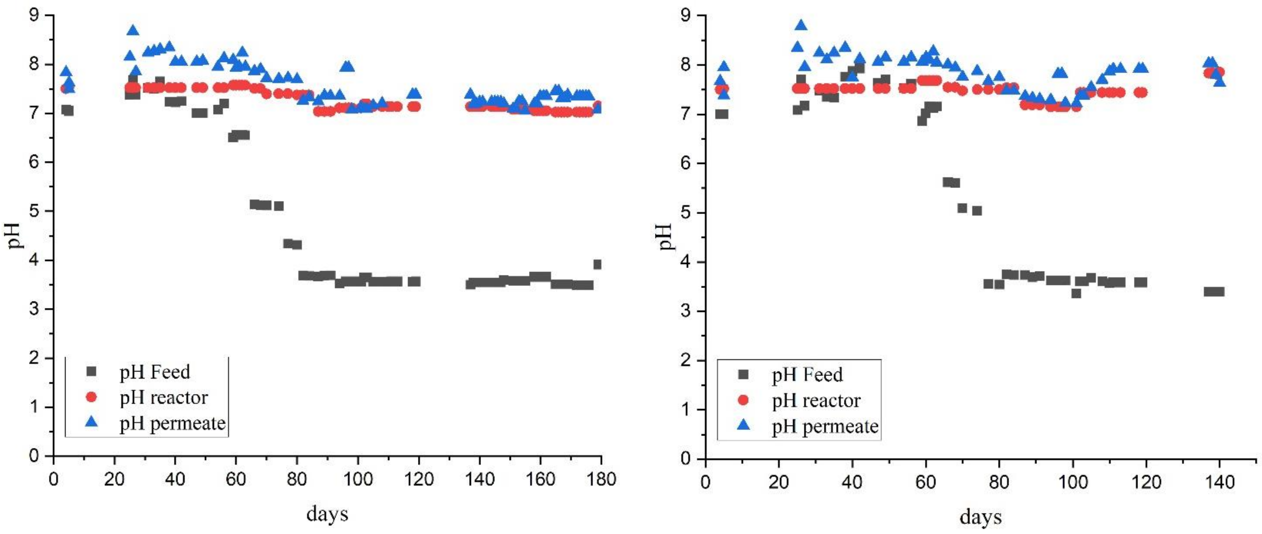

Figure S3. pH monitoring (**A**) AnMBR1-Centrifuged vinasse; (**B**) AnMBR2-Raw vinasse

The alkalinity of a solution is a measure of its ability to neutralize acids, resist pH changes or buffering the system. And this is due to the presence of bases (hydroxides), acid salts weak inorganics (bicarbonate, borate, silicate, phosphate and sulfide) (partial alkalinity), and salts of volatile organic acids (acetate, propionate, butyrate, among others) (intermediary alkalinity) (Ripley et al.,1986). The alkalinity of the reactor is an indication that the anaerobic digestion process occurs adequately. According to Ripley et al. (1986), a ratio between intermediate alkalinity (IA), which is related to the volatile acids, and partial alkalinity (PA), referring bicarbonate alkalinity, lower than 0.3 is indicative for process stability. Commonly, the main evidence of destabilization of methanogenic systems comprises the sudden pH reduction and the significant increase in the CO_2 content in biogas (Ripley et al.,1986). However, pH changes were negligible during the whole reactor operation. A pH drop can only be expected when accumulating VFA consume all alkalinity present in the reaction medium and H^+ ions accumulate (Dilallo and Alberston, 1961).

Table S2. Alkalinity (in mg $CaCO_3.L^{-1}$) during the reactors' operation

		AnMBR1					AnMBR2				
Phase	$gNaHCO_3.g COD^{-1}$	pH	Intermediary alkalinity (IA)	Partial alkalinity (PA)	Total alkalinity	IA/PA	pH	Intermediary alkalinity (IA)	Partial alkalinity (PA)	Total alkalinity	IA/PA
II	0.7	7.9	8,750	4794	13,544	0.55	8.0	11,838	4794	16,631	0.40
II	0.3	8.1	6,988	3039	10,027	0.43	8.3	9,856	3107	12,963	0.31
II	0	7.8	5,258	1349	6,607	0.26	7.9	6,893	1718	8,611	0.25
III	0	7.7	2,697	616	3,313	0.23	7.8	3,114	736	3,849	0.24
IV	0	7.5	2,315	622	2,937	0.27	7.8	3,374	845	4,219	0.25

S4 Volatile Suspended Solids - Experiment 1

The VSS concentration in both reactors was monitored during the reactors' operation. No sludge disposal was performed during the operation with the SRT being approximately equal to the operating time. The VSS concentration gradually increased in both reactors after the adaptation period and the first OLR increase, indicating sludge growth. At the end of the operation the VSS concentration in the AnMBR1 was 39 ± 0.03 g $VSS.L^{-1}$ and in the AnMBR2 was 49 ± 0.02 g $VSS.L^{-1}$.

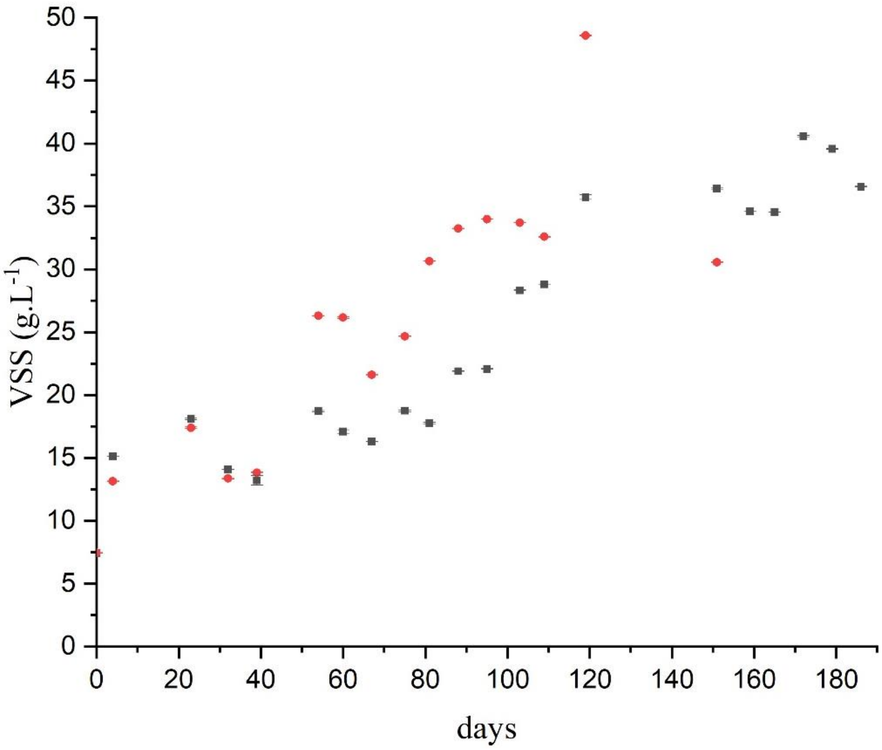

Figure S4. Volatile suspended solids concentration in the AnMBRs during the operation in Experiment 1: ■ AnMBR1; ● AnMBR2, n = 3, bars = standard deviation

S5 Biogas production - Experiment 1

Table S3 presents the biogas production and composition measured during the different stages of the reactors' operation.

Table S3. AnMBR biogas production during Experiment 1 overview.

	AnMBR1 –Centrifuged Vinasse			
Phases	I	II	III	IV
Biogas ($L.day^{-1}$)	6 ± 1	9 ± 2	18 ± 5	27 ± 3
CH4 (%)	49 ± 0.05	52 ± 0.08	47 ± 0.03	47 ± 0.01
CO2 (%)	41 ± 0.001	37 ± 0.08	46 ± 0.03	50 ± 0.01
CH_4 L. g COD $removed^{-1}$	0.29 ± 0.05	0.32 ± 0.05	0.24 ± 0.05	0.29 ± 0.05
	AnMBR2 – Raw Vinasse			
Phases	I	II	III	IV
Biogas ($L.day^{-1}$)	6 ± 2	13 ± 4	18 ± 5	12 ± 6
CH4 (%)	48 ± 0.01	54 ± 0.09	48 ± 0.03	49 ± 0.02
CO2 (%)	43 ± 0.02	38 ± 0.03	48 ± 0.03	48 ± 0.02
CH_4 L. g COD $removed^{-1}$	0.24 ± 0.08	0.28 ± 0.07	0.23 ± 0.04	0.23 ± 0.09

S6 Membrane performance parameters – Experiment 1

Figure S5 shows the membrane parameters monitored during reactor's operation, i.e., filtration resistance (A), TMP (B) and operational permeability (C). The filtration resistance in AnMBR2 started to rise on day 80, concomitant with the flux increase from 4 to 6 LMH. The resistance continued increasing despite the chemical cleaning procedure before the flux was increased to 8 LMH to restore membrane permeability, leading to a complete membrane clogging during Phase IV. The flux was decreased to 4 LMH and the crossflow velocity was increased from 1 $m.s^{-1}$ to 2 $m.s^{-1}$ after performing the membrane cleaning procedure. However, despite the cleaning procedures, the membrane permeability did not recover, and the membrane clogged.

The highest values for the filtration resistance observed in AnMBR1 was at day 120. Subsequently, a chemical cleaning procedure was performed, the CFV was increased to 2 $m.s^{-1}$, and

the system was restarted at a flux of 4 LMH followed by an incrementally raise to 8 LMH. A tentative to increase to 10 LMH resulted in the membrane the clogging of the membrane.

Figure S5. Membrane performance parameters: (**A**) filtration resistance, (**B**) transmembrane pressure, (**C**) permeability

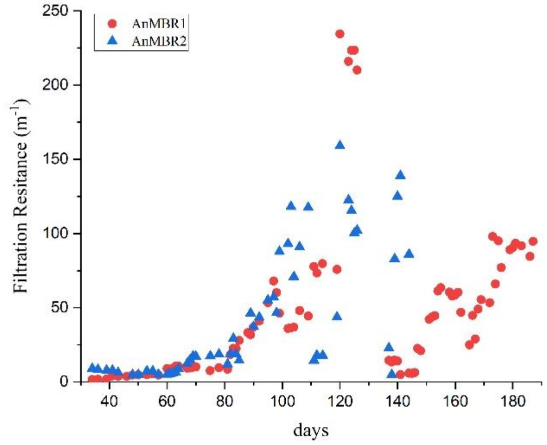

A

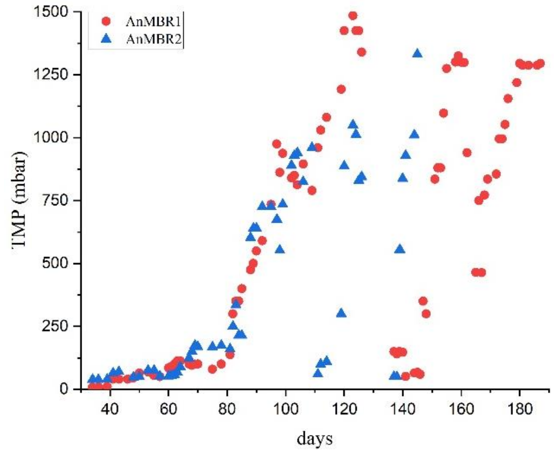

B

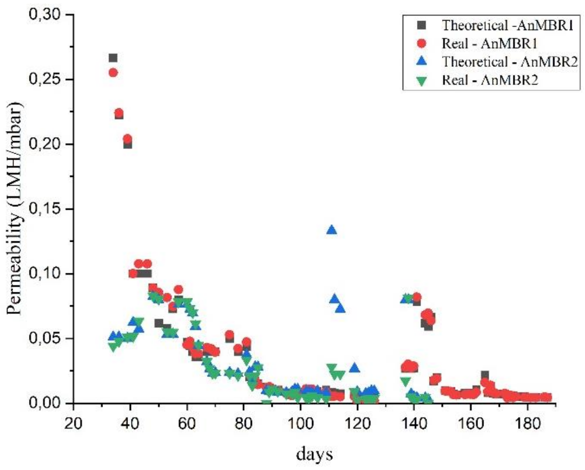

C

S7 Biogas production - Experiment 2

Biogas production and composition obtained in each phase of the second experiment are presented in Table S4. The results are similar to those obtained in the first experiment. Biogas production increased while the CH_4 portion decreased with the OLR increasing.

Table S4. Biogas production during the beet vinasse treatment in AnMBR at the second experiment.

OLR (g COD.L^{-1}.d^{-1})	Biogas (L.d^{-1})	CH_4 (%)	CO_2 (%)	L CH_4. g COD removed $^{-1}$
6	14 ± 3	52 ± 0.02	48 ± 0.02	0.29 ± 0.05
8	19 ± 6	51 ± 0.01	49 ± 0.01	0.31 ± 0.1
10	20 ± 3	48 ± 0.06	52 ± 0.06	0.21 ± 0.08
12	21 ± 4	30 ± 0.03	70 ± 0.03	0.12 ± 0.02
14	25 ± 2	48 ± 0.01	52 ± 0.01	0.20 0.04

S8 Volatile Fatty Acids - Experiment 2

A VFA removal efficiency of 90% was observed in the permeate when compared to the inlet of the AnMBR. The exception occurred after the OLR increased to 10 kg COD. m^{-3}.d^{-1} in phases IV and V (from day 98 to 119), when mainly acetic and propionic acid were accumulated indicating an unbalance in the acetogenic and methanogenic steps. In general, the VFA concentration in the permeate remained below than 100 mg. L^{-1} in almost the entire reactor's operation.

A **B**

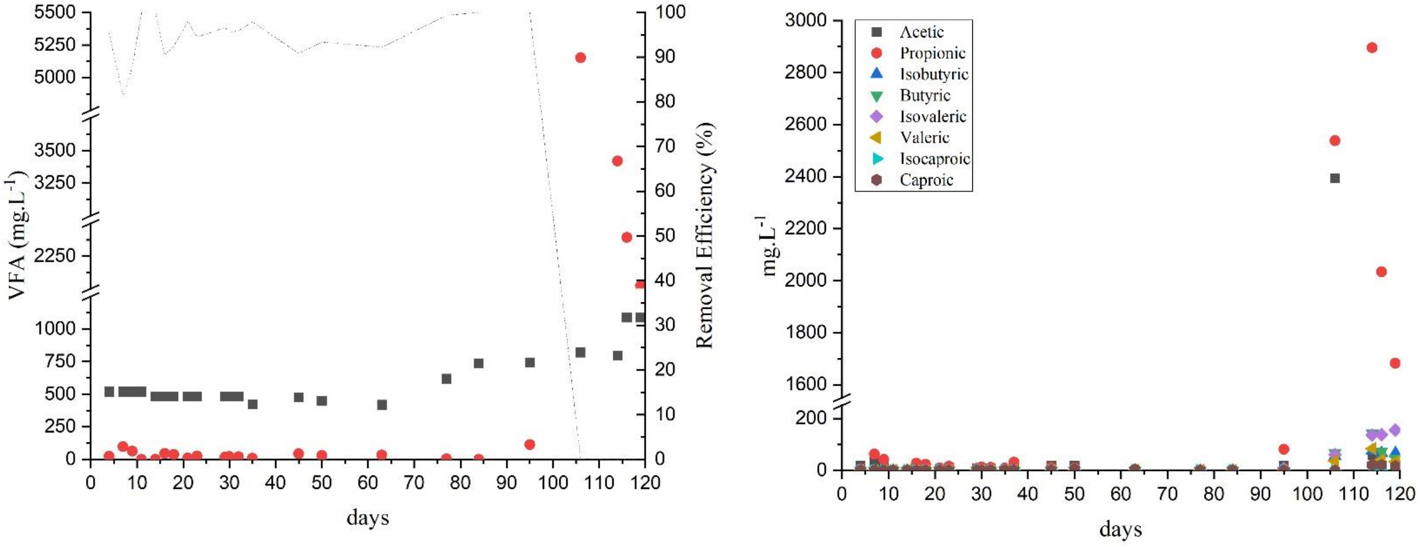

Figure S6. VFA removal efficiency (**A**): ■ Input; ● Output; (···) removal efficiency, and (**D**) VFA accumulation.

References

Dilallo, R., Albertson, O. E. (1961) Volatile acids by direct titration. Journal of Water Pollution Control Federation, 33,356–65. http://www.jstor.org/stable/25034391.

Ripley, L. E., Boyle, W. C., Converse, J. C. (1986). Improved alkalimetric monitoring for anaerobic digestion of high strength wastes. Journal Water Pollution., 58(5), 406–411.
